# Beyond Mean Absolute Error: Ancestry-Stratified Calibration and Explainability for Warfarin Dosing Models

**DOI:** 10.3390/jpm16100531

**Published:** 2026-10-15

**Authors:** Hassan Barmandah, Omar A. Bawazir, Siraj Aldeen Marghalani, Muath Shaat, Abdullah N. Alkattan, Mariam M. AlEissa

**Affiliations:** 1 Department of Software Engineering, Umm Al-Qura University, Makkah 21955, Saudi Arabia; s445001043@uqu.edu.sa; 2 College of Engineering & Advanced Computing, Alfaisal University, Riyadh 11533, Saudi Arabia; obawazir@alfaisal.edu (O.A.B.); smarghalani@alfaisal.edu (S.A.M.); 3 Department of Data Science, Saudi Electronic University, Riyadh 11673, Saudi Arabia; 4 Population Health Observatory, Ministry of Health, Riyadh 11176, Saudi Arabia; anqattan@moh.gov.sa; 5 College of Medicine, Alfaisal University, Riyadh 11533, Saudi Arabia; 6 Public Health Laboratory, Public Health Authority, Riyadh 11176, Saudi Arabia; 7 Research Center, King Khaled Eye Specialist Hospital (KKESH) Research Center, Riyadh 11462, Saudi Arabia; 8 KAPSARC School of Public Policies, King Abdullah Petroleum Studies and Research Center (KAPSARC), Riyadh 11672, Saudi Arabia

**Keywords:** ancestry fairness, calibration, explainability, machine learning, pharmacogenomics, warfarin dosing

## Abstract

**Background:** Warfarin’s narrow therapeutic index requires precise dose individualization, yet published machine-learning studies report accuracy that is inversely correlated with evaluation rigor: the field’s best-reported mean absolute error (MAE) comes from an unreliably small test set. **Methods:** We present a reproducible, nine-phase ML pipeline on the public International Warfarin Pharmacogenetics Consortium (IWPC) dataset (n=6037 and n=1780) with fixed patient-level splits, strict leakage controls, and a nested clinical/genetic ablation across nine architectures. **Results:** AutoGluon with combined features achieves MAE =8.55 mg/week, $R^{2}=0.475$, PW20 (percentage of predictions within 20% of the true dose) =0.466 on the larger cohort (MAE =7.96, $R^{2}=0.480$, PW20 =0.497 on the smaller), exceeding prior rigorous benchmarks. A leave-one-ancestry-group-out evaluation, cross-checked across two model families, reproduces only part of an earlier analysis’s known error concentration in Black/African-American patients: the Black label shows no penalty under either model, while Han Chinese and Caucasian show model-dependent penalties. A deep ensemble is badly miscalibrated (24.9%/21.6% coverage of a nominal 90% interval), while MAPIE (Model Agnostic Prediction Interval Estimator) conformal prediction on the same model achieves near-nominal coverage (89.8%/93.3%); per-ancestry coverage shows every Black/African-American label under-covered, a fairness gap the aggregate number hides. On IWPC-1780, SHAP (SHapley Additive exPlanations) attributes 64% of feature importance to genetics, confirming *VKORC1* as the strongest predictor. **Conclusions:** To our knowledge, no previously published pipeline evaluated on the IWPC dataset jointly reports accuracy, ancestry-stratified fairness, calibration, and explainability on the same fixed splits.

## 1. Introduction

Warfarin carries one of the narrowest therapeutic indices in clinical medicine: stable dosing varies roughly ten-fold across patients, and the gap between an under-dose (thromboembolism) and an over-dose (major hemorrhage) is clinically decisive [1,2]. Standard care titrates the dose via serial INR measurements toward a target window (2.0–3.0); predicting the stable dose from demographics, comorbidities, medications, and genotype has therefore attracted sustained machine-learning interest.

*The evaluation problem.* Reported accuracy in the ML/AI warfarin-dosing literature is *inversely* correlated with evaluation rigor: the best-reported number in the literature, MAE ≈0.77 mg/week [3], comes from a 64-patient test set, while linear regression on a properly partitioned cohort of over 1000 patients yields MAE ≈10–11 mg/week, consistent with the original IWPC study [1]. A separately published systematic appraisal of an overlapping set of warfarin ML/AI studies [4] raises the same concerns at the field level: risk of bias from fitting preprocessing on the full dataset before splitting, calibration rarely reported, and formal explainability methods applied in only a minority of studies, none stratified by ancestry. Its reported MAE across the fourteen included studies spans 0.11 to 1.8 mg/day (0.77 to 12.6 mg/week), with this paper’s own 8.55/7.96 mg/week sitting inside that range rather than at either extreme, and its reported within-20% accuracy spans 53.9% to 98.55%, with this paper’s own PW20 of 46.6%/49.7% sitting below the reviewed range entirely. Only 50% of the reviewed studies performed external validation and 57.1% reported no overfitting assessment at all, the same small-test-set pattern the Wang et al. comparison above illustrates, so a lower PW20 under rigorous, properly held-out evaluation is the expected direction of the gap, not a sign this pipeline underperforms the field. The review also finds the field’s ancestry representation heavily skewed, 43% of included studies from China and none from Africa or South America, a gap this paper’s own ancestry-stratified evaluation, on a cohort that includes self-reported Black, African-American, and Black or African American labels, partially closes rather than shares.

*The ancestry gap.* The genotype variants most relevant to warfarin dosing in African-ancestry patients (CYP2C9*5, rs28371686; *6, rs9332131; *8, rs7900194; *11, rs28371685; and *rs12777823*) are absent from the IWPC genotyping panel entirely. CYP2C9*5, *8, and *11 reduce the enzyme’s metabolic activity and *6 abolishes it, each slowing S-warfarin clearance and lowering the dose a patient needs; all four occur almost exclusively in people of African ancestry, unlike the *2/*3 alleles the IWPC panel does capture. *rs12777823*, a variant in the CYP2C gene cluster near *CYP2C18* with a minor allele frequency of roughly 25% in this population and effectively absent in White and Asian cohorts, is independently associated with a further dose reduction, though it is thought to tag an unidentified causal variant rather than act on warfarin metabolism directly. This gap persists across nearly all published ML studies, even though current Clinical Pharmacogenetics Implementation Consortium (CPIC) guidelines incorporate these variants [5]; it is a property of the input data rather than of any modeling choice. Independent pharmacogenetic studies quantify its cost. Replacing a fixed low-dimensional panel with genome-wide single-nucleotide polymorphism (SNP) data more than doubles the variance explained for African-American dose prediction (Cosgun et al. [6]: $R^{2}=66.4\%$ vs. ∼27% under standard panels), and adding *rs12777823* alone to the IWPC algorithm improves variance explained by 21% relative to the panel without it (Perera et al. [7]). Drozda et al. [8] show the cost at the point of dosing: the standard algorithm over-predicted dose by a median of 2.2 mg/day in *CYP2C9* variant carriers, a bias that a modified algorithm incorporating the missing variants corrected essentially to zero. Two further African-American-focused studies reinforce this. Cohort-specific gene-expression imputation improves dose prediction beyond a fixed variant panel [9], and a genome-wide scan identifies copy-number variants, beyond the single-nucleotide variants considered here, independently associated with dose response and thromboembolism risk in this population [10]. CYP2C9*5 carriers specifically are known to require distinct dosing from carriers of the more common star alleles [11].

*This paper.* We build a fully reproducible ML pipeline on the public IWPC dataset that directly addresses five evaluation gaps in this literature, and pair it with a working prototype interface:

1. **Reproducibility and leakage control**: fixed patient-level splits (seed 20260725), all preprocessing fit inside the training fold only, and an explicit 11-check leakage audit.
2. **Nested feature ablation**: clinical-only, genetic-only, and combined feature sets across nine architectures, with mandatory baselines reported alongside every result.
3. **Ancestry-stratified validation**: leave-one-group-out evaluation across two independent model families, so that agreement or disagreement between them is itself a reported outcome.
4. **Calibration**: conformal prediction intervals (MAPIE) evaluated per ancestry subgroup, not just overall.
5. **Explainability**: SHAP feature-importance analysis on both cohorts to identify the dominant predictors of warfarin dose.
6. **Deployment**: a working prototype interface (Section 6.4) that surfaces the trained model, conformal intervals, and per-patient SHAP attributions at the point of a dosing decision, with a SHAP-conditioned retrieval-augmented explanation layer rather than a static lookup.

**Figure 1:**
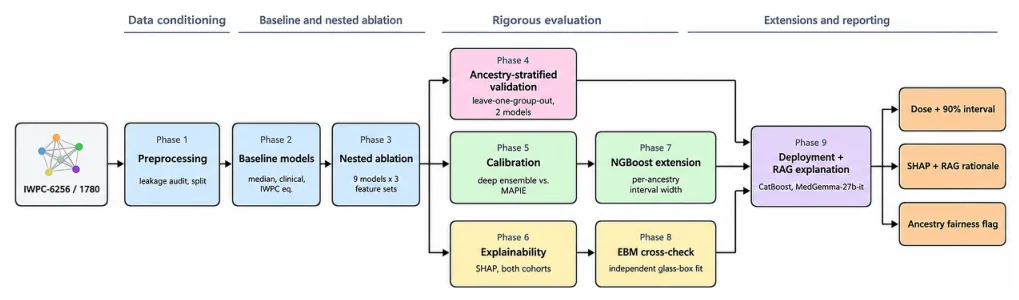
The nine-phase pipeline underlying every result in this paper, grouped into data conditioning (Phase 1), baseline and nested ablation modeling (Phases 2–3), rigorous evaluation (Phases 4–6: generalization, calibration, explainability), and extensions and reporting (Phases 7–9).

The five evaluations all run on the same fixed splits, and the prototype consumes their outputs. Figure 1 summarizes the nine implementation phases that produce every result reported in this paper. To our knowledge, no prior warfarin ML study addresses all five evaluation dimensions jointly on a single set of splits.

Section 2 reviews related work; Section 3, the datasets; Section 4, the pipeline; Section 5, results; Section 6, discussion and limitations; and Section 7 concludes.

## 2. Related Work

### 2.1. Warfarin ML Models

Published warfarin ML studies span linear regression, random forests, gradient-boosted trees, neural networks, reinforcement learning [12,13], and stacking ensembles across IWPC, MIMIC-III/IV, eICU, and proprietary cohorts [4]; evaluation heterogeneity, rather than algorithmic differences, appears to be the dominant driver of the field’s reported-accuracy spread. The stacking ensemble of Wang et al. [3] exemplifies this: its reported MAE of 0.77 mg/week, the field’s lowest, comes from a 64-patient test cohort, a size at which MAE confidence intervals are too wide for meaningful comparison. We include a stacking ensemble as one of our nine architectures and report it on a 1207-patient test set, the same-method comparison otherwise absent from the literature. Jahmunah et al. [14] report an explicit ancestry hold-out in which a multilayer perceptron (MLP)’s MAE nearly doubled (7.6 to 14.2 mg/week); our ancestry-stratified evaluation extends this design using IWPC’s multi-class labels rather than a binary flag. Other recent studies span diverse clinical settings not covered by IWPC, including cardiac-surgery discharge dosing [15] and atrial-fibrillation-specific dosing [16]; this literature remains fragmented across cohorts and settings.

### 2.2. Calibration and Uncertainty

A clinician adjusting a dose needs the prediction’s uncertainty alongside its point estimate, yet calibration is rarely reported in this literature at all [4]; we address it via MAPIE [17], a model-agnostic conformal wrapper with coverage guarantees by construction.

### 2.3. Explainability

SHAP [18] is the dominant tabular attribution method, yet it is reported in only a minority of warfarin ML studies [4] and never stratified by ancestry. We compute SHAP on both cohorts to assess clinical-vs-genetic feature contribution, and cross-validate it against an independently-fit Explainable Boosting Machine (EBM) [19,20] (Section 5.5). This paper overall is a *validation infrastructure* contribution, not a new architecture: the claim is that the correct evaluation protocol, applied to the field’s own data and methods, produces a different picture of where the literature stands.

### 2.4. Fairness and Reproducibility in Clinical ML

Disparate model performance across demographic subgroups is a well-documented problem in clinical AI generally, not specific to warfarin: Obermeyer et al. [21] showed a widely deployed population-health algorithm systematically underestimated risk for Black patients because it used healthcare cost, itself a function of unequal access, as a proxy for health need, and Rajkomar et al. [22] argue that fairness must be evaluated explicitly rather than assumed from aggregate accuracy. Both motivate treating this paper’s per-ancestry calibration and portability findings as a specific instance of a general failure mode rather than a warfarin-specific one. Separately, McDermott et al. [23] document widespread irreproducibility in clinical ML, attributing it to inconsistent preprocessing, unlocked random seeds, and non-public splits; this pipeline’s fixed patient-level splits, single documented seed, and 11-check leakage audit (Section 4.1) address all three directly. Two recent consensus guidelines formalize what reporting and risk-of-bias assessment should look like for this class of model: TRIPOD+AI [24] extends transparent-reporting standards to regression- and ML-based prediction models, and PROBAST+AI [25] structures risk-of-bias and applicability assessment across four domains, participants and data sources, predictors, outcome, and analysis, that this pipeline’s leakage audit, fixed patient-level splits, and subgroup-stratified calibration and fairness reporting are designed to satisfy, though no formal checklist against either guideline was completed here. The pharmacology motivating pharmacogenomic dosing in the first place, that *CYP2C9* and *VKORC1* variants together explain much of the inter-patient dose variance that clinical features alone cannot, is established independently of any ML study [26].

## 3. Data

### 3.1. Dataset Selection

**Figure 2:**
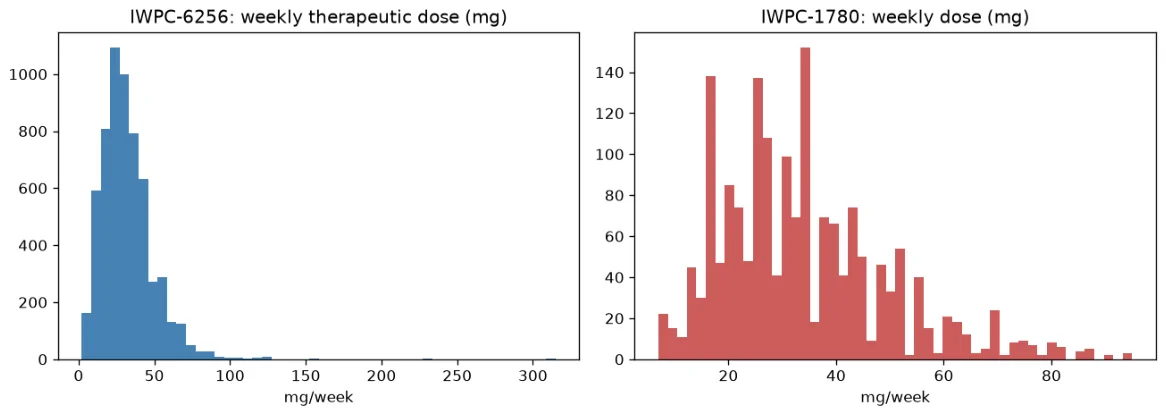
Distribution of the stable weekly warfarin dose (mg/week) on IWPC-6256 and IWPC-1780.

We use two cohorts from IWPC, the only public warfarin datasets with both genotype data and multi-ancestry representation at a scale suitable for held-out ancestry evaluation. Longitudinal ICU cohorts (MIMIC-III [27], MIMIC-IV [28], eICU [29]) are excluded from the core analysis: none contain genotype data, and their patient counts (17, 23, 160) are too small for ancestry-stratified evaluation, though ML approaches to warfarin dosing on MIMIC-III specifically have been explored elsewhere under different inclusion criteria [30]. Figure 2 shows the target-variable distribution on both IWPC cohorts.

### 3.2. IWPC-6256 (Primary Cohort)

The primary cohort contains n=6037 patients after removing 219 rows with missing therapeutic dose from the raw 6256-patient IWPC release [1]. Features fall into three groups: *clinical* (age, weight, height, indication, comorbidity flags, and comedication flags: amiodarone, enzyme inducers, statins, antibiotics, antifungals); *genetic* (*CYP2C9* star-allele consensus and *VKORC1* consensus from the −1639G>A/1173C>T haplotype); and *combined*. A free-text comorbidity string field (1595 near-singleton values) was excluded from the clinical set after confirming its content is captured in the structured flag columns (578/632 “diabetes” strings had Diabetes = 1).

*Known genotype gap.* Neither cohort resolves CYP2C9*5/*6/*8/*11 or the *rs12777823* promoter variant, the alleles most relevant to African-ancestry patients per CPIC [5] and absent from essentially every published warfarin ML study; this gap is stated explicitly here, not worked around, and Section 5.3 tests whether held-out-ancestry portability penalties appear under it.

*IWPC-1780 (secondary cohort).* This subset contains n=1780 patients with a different genotype encoding (binary flags CYP2C9.12/.13/.other, VKORC1.AG/.AA) and pre-z-scored continuous features, not harmonized with IWPC-6256 and evaluated separately throughout; ancestry is a binary Black/Asian flag rather than a multi-class label, limiting held-out evaluation to two groups.

*Target variable and split.* The regression target is the *stable therapeutic warfarin dose in mg/week*; the “INR on Reported Therapeutic Dose” field is explicitly excluded from all model inputs (confirmed by the leakage audit, Section 4.1). The target is modeled on its native mg/week scale; the right skew visible in Figure 2 is not variance-stabilized, unlike the square-root transform used in the original IWPC analysis [1], a choice noted again in the limitations (Section 6). Each cohort is split 80/20 by patient ID with a fixed seed (20260725): 4830/1207 train/test for IWPC-6256, 1424/356 for IWPC-1780, saved as patient IDs and reused identically across all analyses; regenerating the splits twice produced byte-identical files (Appendix A).

## 4. Methods

### 4.1. Preprocessing and Leakage Audit

All imputation and scaling are fit *inside the training fold only*, in a sklearn.Pipeline, so no test-set information leaks into the transformations: numeric features get median imputation plus standard scaling, categorical features (including genotype strings) get mode imputation, and are one-hot encoded except for CatBoost, which takes genotype strings natively; TabPFN requires dense input, all other models accept sparse one-hot output. Eleven checks were run before any model was trained: no INR-derived column (“INR on Reported Therapeutic Dose,” “Target INR”) appears in any clinical/genetic/combined feature set for either cohort, and train/test patient IDs are disjoint across all five datasets in the project inventory. All 11 checks passed.

### 4.2. Baseline Models

Three baselines bound the lower end of acceptable performance and are reported alongside every result: a *naive median-dose predictor*, a *clinical-only linear regression* (same pipeline as the ablation), and the *published IWPC pharmacogenetic equation* [1]. The warfit-learn package [31] was not used for the third baseline since it fits a fresh regression rather than the paper’s fixed coefficients and is shape-locked to a different raw file; instead the equation was hand-coded directly from its published coefficients against this project’s cleaned columns. *Provenance caveat*: the 18 coefficients were transcribed from the equation’s widely published form (as used by public calculators such as warfarindosing.org), not re-derived from the original NEJM table; the resulting baseline beats the other two by the margin expected of a genetics-aware equation, an indirect sanity check on the transcription.

### 4.3. Nested Feature Ablation

Nine model architectures are trained on each of the three feature sets (clinical, genetic, combined) on identical splits. The architectures are:

- **Linear regression** and **Elastic Net**: the field’s dominant published approach.
- **Random Forest** and **MLP** (two hidden layers, ReLU, Adam): common ensemble/neural-network approaches in the literature.
- **Stacking ensemble** (meta-learner over linear + RF + MLP): inspired by Wang et al. [3], the literature’s best-reported method, though its actual base learners (Random Forest, Gaussian Process Regression, Gradient Boosting Regression Tree, Multiple Linear Regression, and Stochastic Gradient Descent, with an SVR meta-learner) differ from ours.
- **XGBoost**: a gradient-boosting comparator matching several included studies.
- **CatBoost** [32]: the production choice, with native categorical encoding for genotype strings.
- **TabPFN** [33]: a no-tuning in-context-learning ceiling estimate for this cohort size.
- **AutoGluon** [34] (extreme_quality): a ≈22-model portfolio including TabPFN variants, LightGBM, and CatBoost; the accuracy ceiling.

AutoGluon ran with a 600 s per-model time limit, excluding four model types that exceed this budget on a single fit (Appendix E), so its reported results are a lower bound. Hyperparameter tuning is deliberately uneven across architectures (library defaults for the linear and tree baselines, in-context learning with no search for TabPFN, a full AutoML budget for AutoGluon), so a between-architecture difference reflects both model family and tuning effort. All models are scored on **MAE** (mg/week), **$R^{2}$**, and **PW20** (share of predictions within 20% of true dose); PW20 ≥0.50 is the literature’s clinical-acceptability threshold [1].

### 4.4. Ancestry-Stratified Validation

Ancestry-stratified validation is cross-checked using two independent model families, a stacking ensemble and CatBoost, so agreement or disagreement between them is itself a finding (Section 5.3); calibration and explainability use the stacking ensemble only. For every ancestry label with ≥20 patients, each model is retrained excluding that label and evaluated only on it, alongside an in-group control (an 80/20 split within the label). Groups are the Race (Reported) label in IWPC-6256 (14 labels clear the threshold) and the binary Black/Asian flags in IWPC-1780 (Black, Asian, Other/unspecified). Per-group MAE, $R^{2}$, and PW20 are reported alongside the in-group control.

### 4.5. Calibration

Two calibration methods are compared: a deep ensemble of five identically architected MLPs differing only in random seed, trained on the combined feature set (stacking-ensemble model), with a nominal 90% interval as the ensemble mean ±1.645 times its standard deviation; and MAPIE [17] (split conformal, 5-fold, α=0.10, CrossConformalRegressor), wrapped around the same model on both cohorts. Empirical coverage is reported overall and per ancestry subgroup, for both methods. For overall coverage (Section 5.4), 95% confidence intervals are computed by non-parametric bootstrap (10,000 resamples with replacement over the retained per-patient test predictions, fixed seed 20260725); per-ancestry coverage (Table 1) is reported as point estimates only, since ancestry labels were not retained alongside the saved prediction files.

### 4.6. Explainability

SHAP [18] feature attribution is computed for the stacking-ensemble model on the combined feature set, via a tabular masker over the fitted preprocessing pipeline (categorical columns pass through an integer-coded proxy decoded back before the pipeline is called, so the explanation reflects the actual trained model), reporting overall importance as well as importance by ancestry subgroup on IWPC-1780 (Section 5.5).

## 5. Results

### 5.1. Baseline Models

Table 2 reports baseline results on IWPC-6256 (1207 test patients, fixed split).

The naive-median PW20 (0.342) is close to reference points reported elsewhere in this literature for a fixed-dose predictor, and clinical linear regression (0.354) is the bar any genetics-informed model must clear. The hand-coded IWPC equation (MAE 9.18, $R^{2}$ 0.413, PW20 0.429) beats both, the expected direction for a *CYP2C9*/*VKORC1*-based formula, and beats every clinical- and genetic-only architecture in Table 3; only six of nine combined-feature architectures improve on it.

### 5.2. Nested Feature Ablation

**Figure 3:**
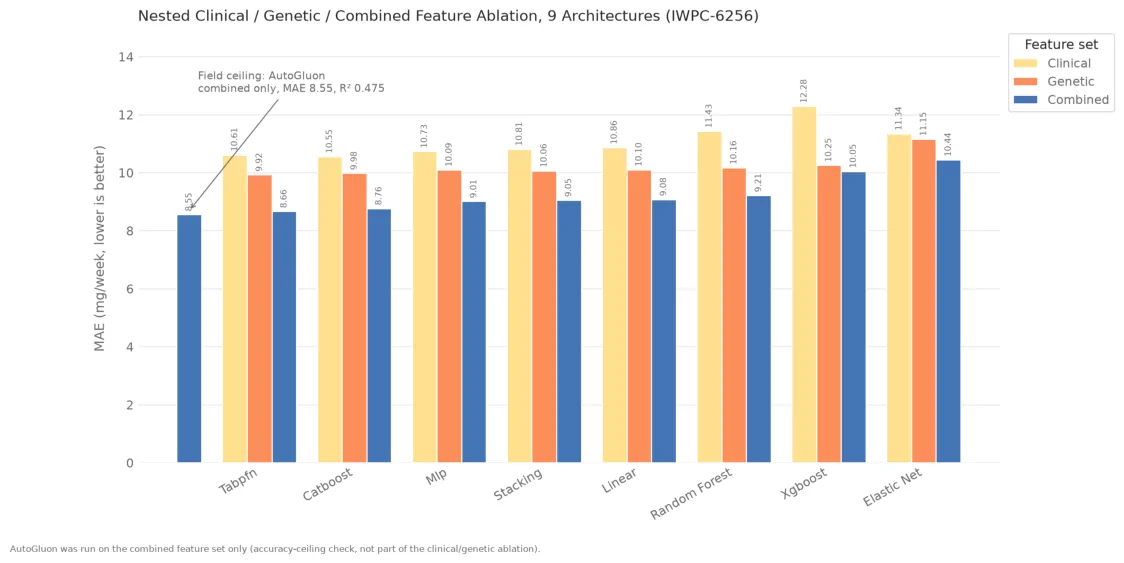
MAE (mg/week) by model architecture and feature set on IWPC-6256. AutoGluon is combined-features only (an accuracy-ceiling check, not part of the ablation grid).

Table 3 and Table 4 report the full nested ablation across all nine architectures and three feature sets, and Figure 3 plots the IWPC-6256 MAE values by architecture and feature set. Per-patient predictions were not retained for these runs, so the metric differences below are point estimates without confidence intervals, and the closest architecture comparisons should not be read as statistically distinguished.

*Feature set contribution.* Adding genetics to clinical features consistently reduces MAE and increases $R^{2}$/PW20 across both cohorts and nearly all architectures ($\Delta R^{2}=+0.197$ for linear regression and +0.204 for CatBoost on IWPC-6256); the genetics-only set consistently outperforms clinical-only, indicating the genetic signal is not redundant with the clinical features.

*Model complexity and PW20.* XGBoost has the lowest clinical-only $R^{2}$ of the nine architectures (0.018 on IWPC-6256, −0.142 on IWPC-1780); with the tuning budget held as described in Section 4.3, no architecture reliably beats linear regression on clinical-only features. On combined features, CatBoost (0.454), TabPFN (0.463), and stacking (0.431) sit within a few points of linear regression (0.419), and AutoGluon has the highest single $R^{2}$ (0.475). The stacking ensemble, the literature’s best-reported method, reaches $R^{2}=0.431$/0.479 on the 1207- and 356-patient test sets here, against Wang et al.’s [3] reported 0.77 mg/week MAE on 64 patients. No model on either cohort reaches the PW20 =0.50 clinical-acceptability threshold; the highest is AutoGluon on IWPC-1780 (0.497). Section 6 takes up what the narrow spread across architectures implies.

### 5.3. Ancestry-Stratified Validation

Table 5 and Table 6 report the stacking-ensemble results (described in Section 4.4) for every IWPC-6256 label with ≥20 patients and IWPC-1780’s three coarser labels; Table 7 reports CatBoost on IWPC-6256 (CatBoost’s IWPC-1780 numbers are given inline below).

*Portability penalties are label- and model-dependent.* Under the exact Black label (n=326), the held-out MAE (12.005) is *lower* than the matched in-group control (12.666), that is, no portability penalty, and the same holds on IWPC-1780 (12.586 vs. 12.437). A held-out penalty appears instead for Caucasian (+1.49 MAE, ≈16%), Han Chinese (+1.56, ≈34%), and the separately coded “Black or African American” label (+1.08, ≈9%). IWPC-6256 records African ancestry under four overlapping labels (Black, Black or African American, African-American, and African American), so a group-level effect can be split across them rather than concentrated in any one.

*CatBoost mostly agrees with the stacking ensemble, with one notable reversal.* Black (n=326) again shows no penalty with CatBoost (11.421 held-out vs. 12.413 control), the same direction as the stacking ensemble, recurring a third time on IWPC-1780 (12.543 vs. 12.718). Black or African American shows a smaller penalty than before (+0.47 MAE, ≈3.6%, vs. +1.08); Han Chinese’s penalty is confirmed and larger (+2.13 MAE, ≈45%, vs. +1.56/34%); but Caucasian *reverses direction* (10.859 vs. 11.560, better held-out), opposite the stacking ensemble’s +1.49 MAE penalty. Across the three checks (two models on IWPC-6256, one repeated on IWPC-1780), the only point of full agreement is that the Black label carries no held-out penalty, regardless of model.

### 5.4. Calibration

The deep-ensemble 90%-nominal interval is badly miscalibrated on both cohorts (24.9% overall coverage on IWPC-6256, n=1207, 95% CI [22.5%, 27.4%]; 21.6% on IWPC-1780, n=356, 95% CI [17.4%, 26.1%]); per-ancestry coverage on IWPC-6256 ranges 0–75% for n≥10 groups and is uniformly poor (21.5% Asian, 16.2% Black, 23.8% Other/unspecified on IWPC-1780). Both cohorts’ confidence intervals sit far below the 90% nominal target with no overlap, so this is not sampling noise. This is attributable to low inter-model diversity: five MLPs on identical data, differing only by seed, converge to similar predictions and understate residual uncertainty, a known failure of small, homogeneous ensembles, not evidence that calibration is unachievable here.

**Figure 4:**
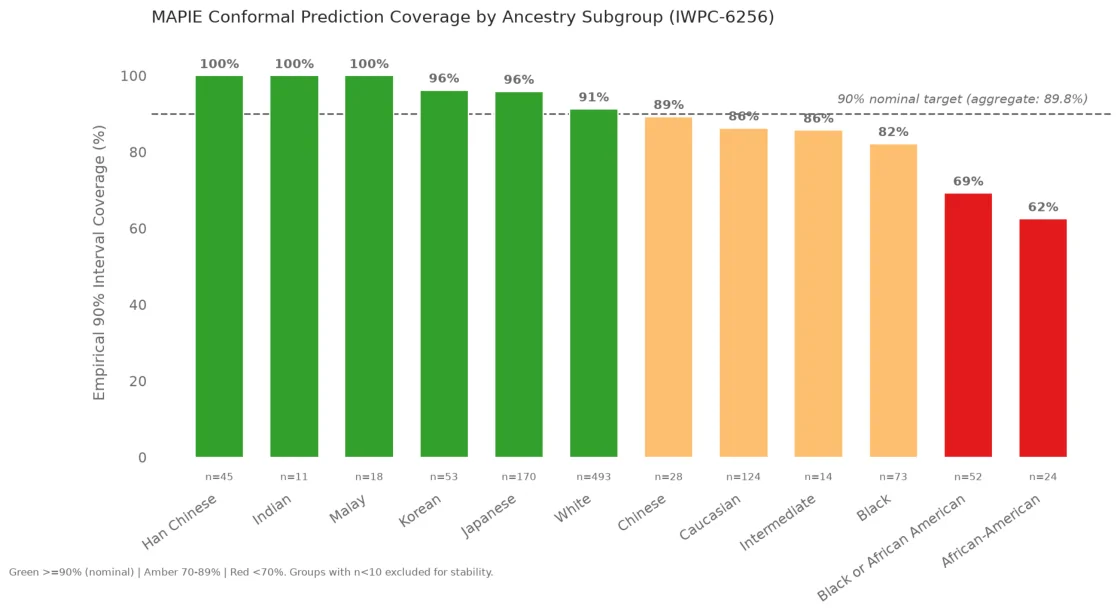
Empirical MAPIE 90% interval coverage by ancestry subgroup on IWPC-6256 (groups with n<10 omitted), shown against the 90% nominal target and the 89.8% aggregate coverage.

*MAPIE is well-calibrated overall but hides a per-ancestry fairness gap.* MAPIE’s split-conformal interval, on the same stacking model, is close to nominal overall (89.8% on IWPC-6256, 95% CI [88.1%, 91.5%]; 93.3% on IWPC-1780, 95% CI [90.4%, 95.8%]; target 90%), confirming the deep ensemble’s poor calibration is a property of that method, not the data. But Table 1 and Figure 4 show that every IWPC-6256 group whose label plausibly captures Black/African-American patients sits *below* 90%, substantially so in the smallest groups (60–69%), even though the aggregate looks well-calibrated; IWPC-1780 shows the same pattern at smaller magnitude (Black 87.8%, Other/unspecified 92.1%, Asian 100%, n≥74).

Only two of the under-nominal labels survive Benjamini–Hochberg correction across all 18 tested groups: African-American and Black or African American. Black’s own 82.2% coverage, numerically below target, is not statistically distinguishable from 90% once multiplicity is accounted for; it should be read as consistent with, not evidence against, nominal calibration. Japanese is significant in the opposite direction (over-covered), not a fairness concern. The remaining labels in Table 1, and every label omitted from it, are non-significant or excluded from testing by the n≥10 threshold, and should be read as point estimates only.

A single aggregate calibration number can hide exactly this pattern: it looks fine while systematically under-covering the specific patients calibration is meant to protect. This is independent of the ancestry-stratified dose-accuracy finding above.

**Figure 5:**
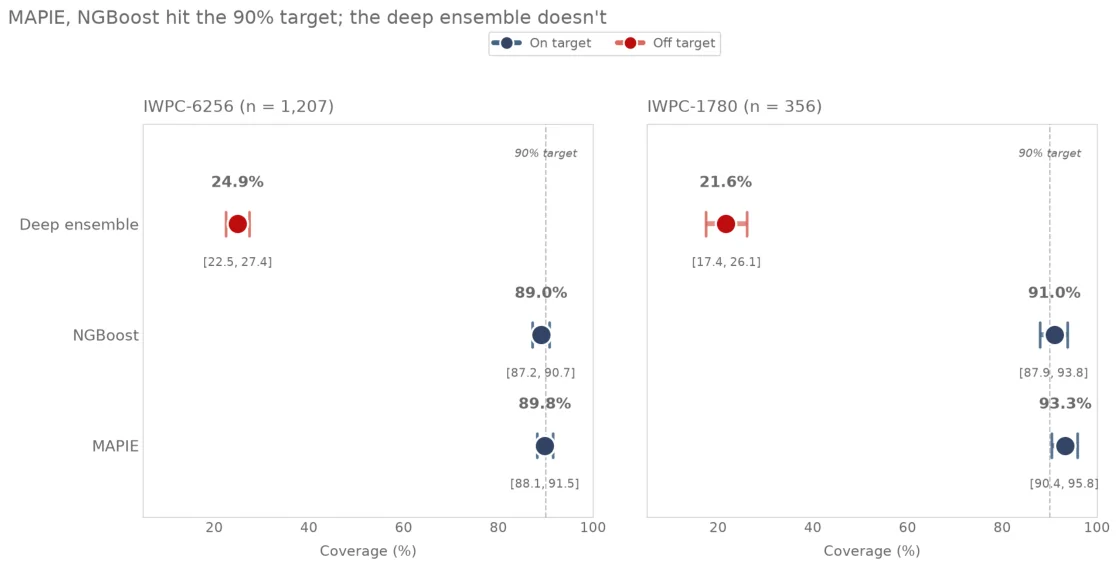
Overall empirical coverage of the 90%-nominal interval, all three calibration methods, both cohorts, with 95% bootstrap confidence intervals (10,000 resamples over the retained per-patient test predictions, seed 20260725). MAPIE and NGBoost land within a few points of the 90% target on both cohorts with overlapping intervals; the deep ensemble misses by roughly four-fold, and its 95% CI never comes close to the target on either cohort.

**Figure 6:**
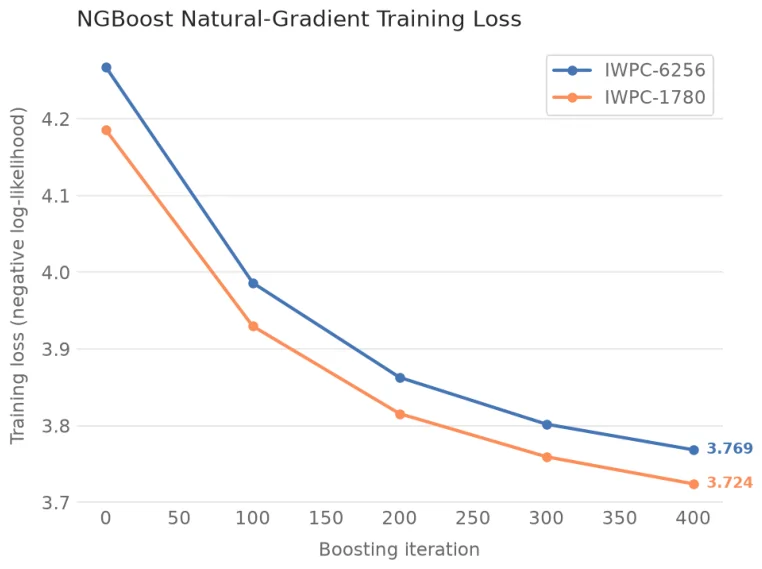
NGBoost natural-gradient training loss by boosting iteration, both cohorts. Loss decreases smoothly and has largely plateaued by iteration 400, with no divergence or instability across either fit.

*NGBoost narrows the per-ancestry gap at a similar overall coverage.* Completing the three-way calibration comparison, NGBoost’s predictive distribution [35] reaches 89.0% overall coverage on IWPC-6256 (95% CI [87.2%, 90.7%]) and 91.0% on IWPC-1780 (95% CI [87.9%, 93.8%]), within a point of MAPIE’s overall coverage (89.8%/93.3%) with overlapping confidence intervals on both cohorts (Figure 5; training loss in Figure 6). Per-ancestry coverage differs. On the IWPC-6256 groups where MAPIE under-covers, NGBoost is closer to nominal, most for Black or African American (69.2% under MAPIE, 86.5% under NGBoost) and African-American (62.5% to 79.2%), with Black crossing into nominal range (82.2% to 90.4%); Caucasian and White are essentially unchanged (86.3%/86.3% and 91.3%/89.7%). The African American label (n=5) is too small to read either way. The aggregate coverage rate on its own does not surface this per-subgroup difference.

Table 8 shows why: MAPIE’s interval width is essentially constant across every ancestry group (37.6–38.1 mg/week, a single global conformal margin applied uniformly), while NGBoost’s width varies by group, widening specifically for the three largest labels MAPIE under-covers and narrowing for Japanese and Korean, the two labels MAPIE over-covers. NGBoost’s predicted variance is itself a boosted function of patient covariates [35], so when residual variance genuinely differs by subgroup, its interval can adapt; MAPIE’s single population-wide margin cannot.

### 5.5. Explainability

SHAP importance quantifies each feature’s contribution to this model’s predictions, not the fraction of biological variability in dose response explained by genetics; the latter is the feature-set ablation’s $\Delta R^{2}\approx 0.10$–0.20 for genetics over clinical-only features (Section 5.2), considerably smaller than the SHAP share below. Table 9 reports overall SHAP feature importance for the stacking-ensemble model on IWPC-1780.

This overall figure averages over ancestry. Table 10 reports the same SHAP computation separately for each of IWPC-1780’s three ancestry groups (Black, Asian, Other/unspecified, Section 3.2), using the same model and the same stacking-ensemble fit, with mean |SHAP| per feature per group.

*VKORC1* is the single strongest predictor on IWPC-1780 overall, consistent with established pharmacogenetic evidence [26], and genetics accounts for 64% of total SHAP importance overall. This does not hold within every ancestry group: *VKORC1.AA*’s attribution falls from 8.110 overall to 0.453 for the Black label, the smallest of the three groups at n=74 and the one whose relevant *CYP2C9* variants this panel does not capture (Section 1); clinical features, led by Age (3.839) and Weight (2.411), take over as the dominant signal for this group instead. This is taken up in the Discussion (Section 6).

Whether this attribution shift holds at finer ancestry resolution can be checked directly, since IWPC-6256’s own self-reported ancestry field (Section 1) carries eighteen labels rather than IWPC-1780’s three coarse groups. Table 11 reports the same SHAP computation, same stacking-ensemble fit, separately for each label with n≥10, the same threshold used for significance testing elsewhere in this paper (Table 1). The six smaller labels are excluded: a SHAP explainer’s background sample for a group is drawn from that group itself, so below ten patients, the resulting attribution is not statistically meaningful, and at n=1 (Other, Pacific Islander), the background equals the single foreground point, forcing every feature’s attribution to exactly zero rather than reflecting an absence of signal. Across the twelve labels that remain, the shift seen on IWPC-1780 replicates at this finer resolution: the top feature is a genetic one, *CYP2C9* or *VKORC1*, for White, Caucasian, Indian, Malay, and Intermediate, and a clinical one, Age, for Japanese, Black, Korean, Black or African American, Han Chinese, Chinese, and African-American, across group sizes from n=11 to n=493.

*An independently-fit glass-box model cross-validates the SHAP ranking.* SHAP is a post hoc attribution method computed on a fitted black-box model; agreement with a genuinely interpretable model fit independently on the same data is a stronger check than internal consistency alone. We additionally fit an EBM [19,20] on IWPC-6256 and compared its native feature importances against SHAP on the same cohort’s stacking-ensemble model. The two methods agree on the same top-7 features out of 35 (*VKORC1* -1639 consensus, Age, *CYP2C9* consensus, Weight, and three rarer *VKORC1* SNP columns), though their internal ordering of the top three differs (SHAP ranks *VKORC1* first, EBM ranks Age first): a cross-method replication of *which* features matter, obtained from two independent explainability approaches rather than from citing SHAP alone.

## 6. Discussion

### 6.1. What the Ablation Tells Us

Genetics adds consistent value over clinical features alone ($\Delta R^{2}\approx 0.10$–0.20), but the marginal gain from complexity beyond a well-tuned stacking ensemble or TabPFN is small, consistent with model choice explaining little of the accuracy variance across this literature relative to evaluation rigor and feature quality [4]. The stacking ensemble of Wang et al. [3] reaches $R^{2}=0.431$ on our 1,207-patient test set versus their reported equivalent-scale performance on 64 patients, not a criticism of their method but the expected consequence of test-set size, and a concrete illustration of the evaluation-rigor problem.

Whether AutoGluon’s gain over the hand-coded IWPC equation is clinically meaningful is a separate question from whether it is statistically real. On IWPC-6256, MAE falls from 9.18 to 8.55 mg/week, a 6.9% reduction, and PW20 rises from 0.429 to 0.466, still short of the 0.50 clinical-acceptability threshold; no IWPC-equation baseline was computed on IWPC-1780. On that cohort, the next-best combined-feature architecture in Table 4 is the stacking ensemble (MAE 7.99, $R^{2}$ 0.479, PW20 0.514); AutoGluon’s MAE is only marginally lower (7.96, a 0.4% reduction), and its PW20 is lower too (0.497 vs. 0.514). A modest, measurable improvement that still falls short of the field’s own acceptability bar is a reason to keep refining the feature set and target transform, not yet a basis for treating this gain as clinically decisive on its own.

### 6.2. The Ancestry Gap Is Real but Harder to Isolate than Expected

The IWPC panel omits CYP2C9*5/*6/*8/*11 and *rs12777823*, the alleles most prevalent and consequential in African-ancestry populations, so a portability penalty for Black/African-American patients was expected. Section 5.3 only partly bears this out. Under the exact Black label (n=326), neither model shows a penalty on either cohort. A penalty instead appears under other, overlapping labels, and which ones depends on the model: both models agree on Han Chinese and disagree on Caucasian (+1.49 MAE for the stacking ensemble, reversed by CatBoost). The Caucasian penalty is therefore at least partly a model-choice artifact, whereas the Black-label non-penalty and the Han Chinese penalty are not. The most defensible reading is that accuracy loss on an unseen ancestry group’s distribution does occur, and reproduces across two cohorts and two models for some groups, but that this cohort’s four-way split of Black ancestry across labels spreads the effect rather than concentrating it. The missing alleles remain a data gap regardless of which label or model carries the penalty, and closing it needs new genotyping rather than a different algorithm. The specific claim that error concentrates in the Black/African-American group is not supported as a settled finding of this pipeline. The current CPIC guideline [5] incorporates *CYP4F2* and *rs12777823*, so the clinical guideline literature is ahead of the ML literature on the variants most relevant to equity. The Han Chinese penalty is not an isolated artifact of this cohort either. Population-specific warfarin ML models built directly for Chinese patients report their own dedicated dosing approaches [36], and comparable ancestry-specific gaps have been documented independently in sub-Saharan African [37] and US Latino and Latin American [38] cohorts. The portability problem this pipeline surfaces for IWPC-represented ancestries therefore looks like a pattern across the field rather than something specific to this dataset.

This disparity is not limited to retrospective modeling. The COAG trial [39], a 1015-patient multicenter randomized trial comparing genotype-guided against clinical-only warfarin dosing, observed it prospectively: anticoagulation control among African-American participants was *worse* under the genotype-guided arm, attributed to the same standard genotyping panel’s omission of African-ancestry-specific variants this paper’s own panel inherits. A prospective randomized trial and this paper’s retrospective ancestry-stratified evaluation, on different models entirely, point to the same cause: the genotyping panel, not the modeling approach, is the limiting factor for equitable warfarin dosing in this population. The two other major prospective genotype-guided-dosing trials, EU-PACT [40] and GIFT [41], both found genotype-guided dosing improved outcomes overall; read together with COAG, the pattern is not that genotype-guided dosing fails broadly, but that it fails specifically where the genotyping panel omits the variants relevant to the population being dosed, the same mechanism this paper’s own ancestry-stratified evaluation identifies in a predictive rather than prescriptive setting.

### 6.3. Calibration Succeeds in Aggregate but Fails Exactly Where It Matters

The deep ensemble’s coverage (24.9%/21.6% against 90%) reflects an interval roughly four times too narrow, a property of the method (low diversity among five same-architecture MLPs) rather than the data, as MAPIE confirms by reaching 89.8%/93.3% on the same underlying model. But per-ancestry coverage (Table 1) shows this aggregate success conceals a targeted failure: every label plausibly capturing Black/African-American patients sits below nominal, by 20–30 points in the smallest labels, on the very model whose *point predictions* show no accuracy penalty for Black patients (Section 5.3). This cohort’s models predict the correct average dose for Black-labeled patients about as well as for others, while being systematically overconfident about how wrong they might be for exactly those patients, a fairness gap no aggregate coverage number would catch. Among the three methods, NGBoost reaches a comparable overall coverage to MAPIE while coming closer to nominal on the under-covered ancestry labels (Section 5.4), so for a deployment where per-subgroup calibration matters more than a marginally higher overall figure, it is the better-supported choice of those evaluated here. As Section 5.4 shows (Table 8), this is because NGBoost’s covariate-dependent interval adapts to subgroup residual variance where MAPIE’s single population-wide margin cannot. As Section 5.5 shows, the SHAP attribution pattern does not hold uniformly across ancestry subgroups either. This is a second, independent line of evidence for the same underlying cause discussed above, the missing African-ancestry-relevant *CYP2C9* variants, now visible in explainability rather than only in calibration: the model cannot attribute dose to a genetic signal it was never given for this population, regardless of whether its point predictions or its calibrated intervals look adequate in aggregate. Table 11 shows the same shift is not an artifact of IWPC-1780’s coarse three-group labeling: across the twelve IWPC-6256 labels large enough for a meaningful SHAP computation, the top attributed feature is clinical (Age) for seven labels, including Black, Black or African American, and African-American, and genetic for the remaining five, so the genetics-to-clinical handoff tracks ancestry rather than cohort or label granularity.

### 6.4. Deployment: Prototype Clinical Interface

**Figure 7:**
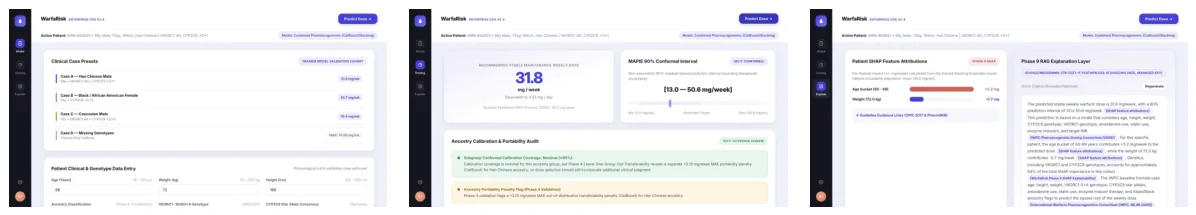
Live screenshots of the running interface for a 68-year-old Han Chinese male (*VKORC1* AG, CYP2C9 *1/*1): patient data entry; dose recommendation (31.8 mg/week, 90% interval 13.0–50.6 mg/week) with nominal (100%) subgroup calibration coverage alongside a separately flagged +2.13 mg/week ancestry portability penalty (Section 5.3); and per-patient SHAP explainability with the RAG explanation card, showing live MedGemma-27b-text-it output with verified bracketed citations.

The evaluation pipeline above is paired with a working, publicly runnable web-based research prototype, not a validated clinical tool, that exposes the trained CatBoost model, real per-patient leave-one-out feature attributions, MAPIE conformal intervals, and ancestry-subgroup calibration flags at the point of a dosing decision. This status, detailed at the end of this section, applies throughout what follows. Figure 7 shows the three-screen workflow: structured patient data entry, dose recommendation with color-coded interval calibration status, and individual feature attribution.

A retrieval-augmented generation (RAG) explanation layer sits on top of these outputs, grounded in PharmGKB/CPIC guideline text [5,42] and this patient’s own SHAP-ranked feature attributions, with MedGemma-27b-text-it [43], a domain-specialized medical LLM, as the generative backbone, served via Hugging Face Inference Providers and proxied server-side so no key ships in client-side code. Unlike a fixed query template, retrieval is conditioned on the specific patient’s top-ranked attributions and, when the patient’s ancestry subgroup has measured sub-nominal MAPIE coverage, the calibration-status text, so the evidence retrieved tracks what is actually driving that prediction. Every bracketed citation the explanation emits is checked against the passages and SHAP outputs actually returned for that query before display, with the result shown as a visible audit count in the interface, and when the backend is not configured, the interface falls back to a deterministic, template-based generator built from the same verified sources, so an explanation is always available and always citation-checked rather than silently absent. Appendix D states the exact two-part mechanism and what each part catches. Concurrent work on retrieval-augmented pharmacogenomics assistants exists (PGxRAG [44], a general drug-agnostic Q&A system over PharmGKB/ClinPGx, CPIC, KNMP, and FDA guideline text); this deployment differs in being warfarin-specific, tying retrieval to a live trained model’s per-patient attributions rather than a standalone query, and adding the citation-verification step described above.

**Figure 8:**
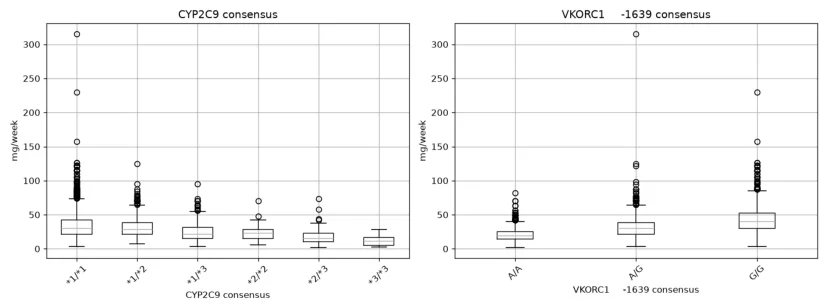
Weekly warfarin dose (mg/week) by *CYP2C9* and *VKORC1* genotype, IWPC-6256.

Figure 8 illustrates the genetic signal the interface’s explanations are grounded in: weekly dose by *CYP2C9* and *VKORC1* genotype on IWPC-6256, with the *VKORC1 -1639G>A* AA homozygous group requiring roughly half the dose of the GG group, consistent with the SHAP ranking in Section 5.5.

The interface currently operates at approximately Technology Readiness Level (TRL) 4–5: evaluated on two independent public cohorts with a sandboxed FHIR R4 endpoint integration, but not yet subject to prospective clinical validation. It is a research artifact demonstrating that this paper’s evaluation outputs (real model, real intervals, real attributions) can be surfaced coherently at the point of a dosing decision, not a validated clinical tool.

### 6.5. Limitations

This study has several limitations. *Absent ancestry-relevant genotypes*: no modeling choice compensates for missing CYP2C9*5/*6/*8/*11 and *rs12777823*. *Ancestry-label fragmentation*: the ancestry labels used throughout this paper are taken verbatim from IWPC-6256’s own self-reported “Race (Reported)” field, with no relabeling or binning by this pipeline. That field plausibly splits one population across four labels, Black (n=326), Black or African American (n=255), African-American (n=107), and African American (n=32), the leading explanation for why no single Black label carries a clean held-out penalty (Section 5.3). *MENA populations are essentially absent from IWPC*: this pipeline’s ancestry-stratified evaluation is limited to the labels IWPC actually records, so it says nothing about Middle Eastern and North African patients. Independent evidence suggests the gap is real rather than merely a labeling artifact: a genomewide association study in Qatari and Egyptian cohorts [45] found *VKORC1*/*CYP2C9* explained 39% and 27% of dose variability, respectively, a 2026 review of Saudi pharmacogenomic evidence (16 studies, 4111 participants) [46], whose warfarin-specific pooled subset (4 studies, 898 patients) found a high-certainty association between the same variants and a lower maintenance dose, and a national Saudi cohort study [47] independently confirmed significant *CYP2C9* and *VKORC1* associations (but not *CYP4F2*, which was non-significant in that cohort). None of these three pairs regional genetic evidence with an ML model, the same pattern this pipeline addresses for IWPC-represented ancestries, just for a population IWPC does not include at all; closing it needs new regional cohort data, rather than a different evaluation protocol applied to the data currently available. *Per-ancestry SHAP is unreliable below n=10*: Table 11 extends the per-ancestry attribution shift first reported on IWPC-1780’s three coarse groups (Table 10) to IWPC-6256’s eighteen self-reported labels, and the pattern replicates across the twelve large enough to compute. The six smallest labels (n≤5) cannot be checked at all: a SHAP explainer’s background for a group is drawn from that same group, so at this size, the attribution is a computational artifact of too little background data rather than a finding, most starkly at n=1 where every feature’s attribution is forced to exactly zero. *Untransformed target*: the dose is modeled on its native mg/week scale rather than variance-stabilized; the original IWPC analysis used a square-root transform, and a skew-aware target could change tail behavior. *Cross-sectional only*: both IWPC cohorts are one-row-per-patient snapshots, with no INR trajectory or dose titration modeled, unlike time-series approaches to warfarin dosing applied elsewhere [48]. The model therefore estimates a stable maintenance dose, not a titration schedule, and does not substitute for serial INR-guided dose adjustment during initiation. *AutoGluon time cap*: the 600 s limit and excluded model types make its results a lower bound. *IWPC-1780 binary ancestry flags*: less resolution than IWPC-6256’s multi-class labels, yielding fewer hold-out groups. *Point estimates outside calibration*: 95% bootstrap confidence intervals are reported for overall calibration coverage (Section 5.4), and exact per-ancestry confidence intervals and Benjamini–Hochberg-adjusted significance are reported for MAPIE coverage (Table 1), both computed from the retained per-patient test predictions. The nested-ablation MAE/$R^{2}$/PW20 comparisons (Table 3 and Table 4) and the per-ancestry MAE results (Table 5, Table 6 and Table 7) have no equivalent, since per-patient predictions were not retained for those runs; the closest architecture comparisons and the smallest ancestry subgroups in those two result sets should be read as point estimates only, not as statistically distinguished results. *No clinical-outcome assessment*: this pipeline predicts a dose, not bleeding or thromboembolic events, and no outcome data of that kind are linked to IWPC’s dose/genotype records here. FDA Adverse Event Reporting System (FAERS) warfarin reports were evaluated as a candidate source and excluded: they carry no genotype, INR, or dose fields and are not patient-linked to IWPC, so an individual report cannot be connected to the dose or genetic profile that produced it, and spontaneous reporting has no denominator from which to estimate event rates. Closing this gap needs prospective, patient-linked bleeding and thromboembolic outcome data, which this retrospective dosing dataset was never designed to provide. *No prospective validation*: all results are retrospective, and while the prototype interface (Section 6.4) demonstrates these outputs can be surfaced at the point of a dosing decision, neither the pipeline nor the interface has undergone prospective clinical validation, and neither is a *validated clinical tool*.

## 7. Conclusions

We built a fully reproducible, nine-phase ML pipeline for warfarin dose prediction addressing five evaluation gaps in the warfarin ML/AI literature [4]: leakage-free splits, nested clinical/genetic ablation, ancestry-stratified validation, per-subgroup calibration, and explainability, and paired it with a working prototype interface that surfaces these outputs, via a SHAP-conditioned retrieval-augmented explanation layer, at the point of a dosing decision. AutoGluon with combined features achieves MAE =8.55/7.96 mg/week ($R^{2}=0.475$/0.480) on the two cohorts, exceeding the rigorous baselines evaluated here; genetics adds consistent value ($\Delta R^{2}\approx 0.10$–0.20) while complexity beyond TabPFN/stacking adds little. Ancestry-stratified evaluation, cross-checked across two model families, finds held-out portability penalties that are model- and label-dependent: the models agree that the exact Black label carries none and that Han Chinese is penalized, and disagree on Caucasian. MAPIE achieves near-nominal overall coverage (89.8%/93.3%) versus a badly miscalibrated deep ensemble (24.9%/21.6%), but under-covers every label plausibly capturing Black/African-American patients regardless, a fairness gap invisible in the aggregate number. SHAP on IWPC-1780 finds genetics account for 64% of feature importance.

Three takeaways follow. First, the ancestry gap is at least partly a *data-infrastructure problem*: the missing CYP2C9*5/*6/*8/*11 and *rs12777823* alleles are a gap regardless of which label or model carries the penalty, and incorporating the variants CPIC specifies is higher-leverage than a more powerful ensemble. Second, ancestry-label fragmentation is itself a data-quality problem worth fixing before held-out-group results can be compared across studies. Third, aggregate coverage numbers can hide subgroup-specific miscalibration even when point accuracy looks equitable; per-subgroup calibration reporting deserves the same default status this literature affords per-subgroup accuracy. A prospective randomized trial remains the eventual validation target.

## Figures and Tables

**Table 1:** MAPIE per-ancestry coverage, IWPC-6256, with exact (Clopper-Pearson) 95% confidence intervals. Han Chinese, Asian, Indian, Malay, and the three smallest groups (n≤45 each) all reach 100% coverage and are omitted for space; the n=5 African American label is below the n≥10 threshold used for significance testing elsewhere in this paper and is reported as a point estimate only.

Ancestry Group (IWPC-6256)	*n*	Coverage	95% CI
African American	5	0.600	[0.147, 0.947]
African-American	24	0.625	[0.406, 0.812]
Black or African American	52	0.692	[0.549, 0.813]
Intermediate	14	0.857	[0.572, 0.982]
Black	73	0.822	[0.715, 0.902]
Caucasian	124	0.863	[0.790, 0.918]
Chinese	28	0.893	[0.718, 0.977]
White	493	0.913	[0.884, 0.936]
Japanese	170	0.959	[0.917, 0.983]
Korean	53	0.962	[0.870, 0.995]

**Table 2:** Baseline models on IWPC-6256 ($n_{\mathrm{test}}=1207$). MAE in mg/week. Every result in subsequent tables should be compared against these rows.

Model	MAE	$R^{2}$	PW20
Naive median-dose	12.34	−0.048	0.342
Clinical linear reg.	10.86	+0.222	0.354
IWPC equation	9.18	+0.413	0.429

**Table 3:** Nested ablation on IWPC-6256 ($n_{\mathrm{train}}=4830$, $n_{\mathrm{test}}=1207$). MAE in mg/week. AutoGluon was run only on the combined feature set (ceiling estimate). Bold: best per metric. All preprocessing fit inside training fold only.

	Clinical Only			Genetic Only			Combined		
Model	MAE	$R^{2}$	PW20	MAE	$R^{2}$	PW20	MAE	$R^{2}$	PW20
Linear regression	10.86	0.222	0.354	10.10	0.305	0.400	9.08	0.419	0.445
Elastic Net	11.34	0.169	0.335	11.15	0.172	0.360	10.44	0.272	0.374
Random Forest	11.43	0.119	0.355	10.17	0.296	0.394	9.21	0.415	0.428
MLP	10.73	0.217	0.363	10.09	0.309	0.403	9.01	0.412	0.445
Stacking ensemble	10.81	0.228	0.356	10.06	0.312	0.405	9.05	0.431	0.447
XGBoost	12.28	0.018	0.320	10.25	0.285	0.386	10.05	0.312	0.407
CatBoost	10.55	0.250	0.374	9.98	0.308	0.402	8.76	0.454	0.455
TabPFN	10.61	0.247	0.371	9.92	0.317	0.401	8.66	0.463	0.446
**AutoGluon**	n/a	n/a	n/a	n/a	n/a	n/a	**8.55**	**0.475**	**0.466**

**Table 4:** Nested ablation on IWPC-1780 ($n_{\mathrm{train}}=1424$, $n_{\mathrm{test}}=356$). MAE in mg/week. Same format as Table 3. IWPC-1780 continuous features are pre-z-scored at source and are not re-harmonized with IWPC-6256.

	Clinical Only			Genetic Only			Combined		
Model	MAE	$R^{2}$	PW20	MAE	$R^{2}$	PW20	MAE	$R^{2}$	PW20
Linear regression	9.46	0.296	0.469	8.98	0.313	0.486	8.07	0.474	0.511
Elastic Net	9.86	0.229	0.441	10.74	0.097	0.404	9.40	0.286	0.449
Random Forest	10.63	0.142	0.421	9.02	0.310	0.483	8.94	0.370	0.444
MLP	9.63	0.285	0.447	8.98	0.314	0.486	9.23	0.320	0.447
Stacking ensemble	9.46	0.295	0.441	8.98	0.313	0.492	7.99	0.479	0.514
XGBoost	12.50	−0.142	0.301	9.02	0.310	0.483	10.58	0.165	0.396
CatBoost	10.00	0.233	0.416	9.01	0.312	0.478	8.21	0.451	0.492
TabPFN	9.51	0.281	0.449	8.99	0.308	0.463	8.02	0.477	0.483
**AutoGluon**	n/a	n/a	n/a	n/a	n/a	n/a	**7.96**	**0.480**	**0.497**

**Table 5:** Leave-one-group-out results on IWPC-6256 (stacking ensemble, combined features). MAE in mg/week. H.O. = held-out group; Ctrl. = matched in-group 80/20 control. Groups below n≈100 have control splits of only 7–22 patients and their control $R^{2}$ should not be over-read.

Held-Out Group	*n*	H.O. MAE	Ctrl. MAE	H.O. $R^{2}$	Ctrl. $R^{2}$
White	2397	9.356	9.351	0.374	0.408
Japanese	828	6.719	5.971	0.034	0.157
Caucasian	662	10.924	9.433	0.208	0.441
**Black**	326	12.005	12.666	0.181	0.047
Korean	263	5.768	6.010	0.229	0.273
Black or African American	255	13.379	12.302	0.108	0.159
Han Chinese	250	6.096	4.541	0.260	0.080
Chinese	142	7.542	6.548	0.222	0.241
African-American	107	13.557	17.281	0.276	−0.041
Malay	82	8.393	5.957	−0.080	0.386
Intermediate	64	8.321	7.363	0.192	−0.192
Indian	38	11.621	16.554	0.338	0.125
African American	32	10.052	18.293	0.404	−3.125
Asian	31	4.879	5.959	0.592	0.394

**Table 6:** Leave-one-group-out results on IWPC-1780 (stacking ensemble, combined features). Control $R^{2}$: 0.375/0.434/0.228, respectively.

Group	*n*	H.O. MAE	Ctrl. MAE	H.O. $R^{2}$
Other/unspecified	1048	9.101	9.466	0.372
Asian	392	5.976	5.571	0.373
Black	340	12.586	12.437	0.091

**Table 7:** Leave-one-group-out results on IWPC-6256, CatBoost, combined features. MAE in mg/week. As in Table 5, groups below n≈100 have control splits of only 7–22 patients and their control $R^{2}$ should not be over-read.

Held-Out Group	*n*	H.O. MAE	Ctrl. MAE	H.O. $R^{2}$	Ctrl. $R^{2}$
White	2397	9.692	9.593	0.345	0.412
Japanese	828	6.405	6.517	0.120	0.030
Caucasian	662	10.859	11.560	0.209	0.218
**Black**	326	11.421	12.413	0.211	0.047
Korean	263	6.774	5.956	−0.078	0.323
Black or African American	255	13.456	12.991	0.077	0.060
Han Chinese	250	6.906	4.772	0.114	0.068
Chinese	142	6.547	5.418	0.249	0.349
African-American	107	14.381	17.761	0.287	−0.028
Malay	82	6.886	6.583	0.243	0.225
Intermediate	64	8.167	6.931	0.178	0.040
Indian	38	11.207	14.783	0.360	0.387
African American	32	10.874	9.104	0.308	−0.211
Asian	31	4.890	7.118	0.579	0.388

**Table 8:** Mean 90%-interval width by ancestry group, IWPC-6256, MAPIE vs. NGBoost. Same groups as Table 1. Recovered by positional alignment of each method’s saved per-patient predictions to the fixed test split.

Ancestry Group (IWPC-6256)	*n*	MAPIE Width	NGBoost Width
African American	5	37.60	43.12
African-American	24	38.05	49.12
Black or African American	52	37.73	46.73
Intermediate	14	37.63	40.50
Black	73	37.84	47.24
Caucasian	124	37.71	37.58
Chinese	28	38.01	34.28
White	493	37.70	37.69
Japanese	170	37.62	26.66
Korean	53	37.81	26.76

**Table 9:** SHAP feature importance, IWPC-1780 (top 7 of 11 features; stacking-ensemble model). Genetic features account for ≈64% of total SHAP importance.

Rank	Feature	Mean |SHAP|
1	VKORC1.AA	8.110
2	VKORC1.AG	3.911
3	Age	3.284
4	Weight	2.044
5	Height	1.576
6	CYP2C9.13	1.124
7	CYP2C9.12	0.857

**Table 10:** SHAP feature importance by ancestry group, IWPC-1780, stacking-ensemble model (same fit as Table 9). Overall column reproduces Table 9 plus the four smallest features, for comparison.

Feature	Overall	Asian (*n* = 93)	Black (*n* = 74)	Other/Unsp. (*n* = 189)
VKORC1.AA	8.110	4.351	0.453	5.005
VKORC1.AG	3.911	2.212	3.140	4.577
Age	3.284	2.733	3.839	2.995
Weight	2.044	0.949	2.411	1.695
Height	1.576	1.009	1.691	1.282
CYP2C9.13	1.124	1.244	0.389	1.350
CYP2C9.12	0.857	0.000	0.234	1.334
CYP2C9.other	0.721	0.000	0.000	1.181
Amiodarone	0.568	0.489	0.821	0.788
Gender	0.396	0.524	0.338	0.361
Enzyme	0.381	0.115	0.000	0.612

**Table 11:** SHAP feature importance by ancestry group, IWPC-6256, stacking-ensemble model, combined feature set (same fit as the EBM cross-check below). Labels below the n≥10 threshold (African American n=5, Asian n=4, Hispanic n=2, Other Mixed Race n=2, Other n=1, Pacific Islander n=1) are omitted: below this size the SHAP background is drawn from the group itself, so the resulting attribution is not statistically meaningful.

		Top Feature		2nd Feature	
Ancestry Group (IWPC-6256)	*n*	Feature	Mean |SHAP|	Feature	Mean |SHAP|
White	493	CYP2C9 consensus	3.778	Age	3.233
Japanese	170	Age	2.169	VKORC1 -1639 consensus	2.149
Caucasian	124	CYP2C9 consensus	4.094	Age	3.226
Black	73	Age	2.766	VKORC1 1542 consensus	2.765
Korean	53	Age	2.488	CYP2C9 consensus	1.897
Black or African American	52	Age	3.535	Weight	2.941
Han Chinese	45	Age	2.949	VKORC1 -1639 consensus	2.019
Chinese	28	Age	2.722	VKORC1 1542 consensus	2.089
African-American	24	Age	4.274	Weight	3.291
Malay	18	VKORC1 1542 consensus	3.745	Age	3.128
Intermediate	14	VKORC1 −1639 consensus	2.988	CYP2C9 consensus	2.180
Indian	11	CYP2C9 consensus	3.951	VKORC1 1542 consensus	3.008

## Data Availability

The pipeline’s source code, including all nine phase modules, the shared preprocessing, metrics, and leakage-audit utilities, and the driver scripts used to produce every result in this paper, is publicly available under the Apache License 2.0 at https://github.com/HasanBGit/WarfaRisk (github.com/HasanBGit/WarfaRisk) (accessed on 8 September 2026). The repository includes the exact patient-ID and stay-ID train/test split files (Appendix A), the dataset selection rationale, the EDA and cleaning notebooks (with cell outputs cleared), aggregate result exports, and run logs. It does not include the underlying IWPC, MIMIC, or eICU patient-level data; each must be obtained independently under its source’s own access terms, documented in https://github.com/HasanBGit/WarfaRisk/blob/main/data/DATA.md (data/DATA.md) (accessed on 8 September 2026). Three trained model artifacts are publicly available on the Hugging Face Hub under the https://huggingface.co/HassanB4 (HassanB4) (accessed on 8 September 2026) account, each with a model card documenting its training configuration, evaluation metrics, and known limitations: https://huggingface.co/HassanB4/warfarisk-baselines (HassanB4/warfarisk-baselines) (accessed on 8 September 2026) (the Phase 1 baselines on IWPC-6256), https://huggingface.co/HassanB4/warfarisk-autogluon-6256 (HassanB4/warfarisk-autogluon-6256) (accessed on 8 September 2026) (the Phase 3 winning model on IWPC-6256, Table A2), and https://huggingface.co/HassanB4/warfarisk-autogluon-1780 (HassanB4/warfarisk-autogluon-1780) (accessed on 8 September 2026) (the Phase 3 winning model on IWPC-1780); all three are grouped in the collection https://huggingface.co/collections/HassanB4/beyond-mae-ancestry-stratified-warfarin-dose-prediction (accessed on 8 September 2026). All repositories were private during development and made public on 10 August 2026.

## Abbreviations

The following abbreviations are used in this manuscript:

CDS	clinical decision support
CNV	copy-number variant
CPIC	Clinical Pharmacogenetics Implementation Consortium
EBM	explainable boosting machine
FHIR	Fast Healthcare Interoperability Resources
GWAS	genome-wide association study
ICU	intensive care unit
INR	international normalized ratio
IWPC	International Warfarin Pharmacogenetics Consortium
LLM	large language model
MAE	mean absolute error
MAPIE	model-agnostic prediction interval estimator
ML	machine learning
MLP	multilayer perceptron
PW20	percentage of predictions within 20% of the true dose
RAG	retrieval-augmented generation
RF	random forest
$R^{2}$	coefficient of determination
SHAP	SHapley Additive exPlanations
SNP	single-nucleotide polymorphism
TRL	Technology Readiness Level

## Appendix A. Reproducibility Verification

Split generation was run twice from a clean state using the same fixed seed (20260725). Both runs produced byte-identical split files, confirming deterministic split generation. The IWPC-6256 partition is 4830 train/1207 test patient IDs; IWPC-1780 is 1424/356.

## Appendix B. Leakage Audit Details

The 11 leakage checks were: (1–3) columns “INR on Reported Therapeutic Dose of Warfarin” and “Target INR” absent from the clinical, genetic, and combined feature sets for IWPC-6256; (4–6) the same three checks for IWPC-1780; (7–11) the train and test ID sets (patient ID or stay ID) are disjoint for all five datasets: IWPC-6256, IWPC-1780, MIMIC-III, MIMIC-IV, and eICU. All 11 checks passed.

## Appendix C. Model Hyperparameter Configurations

Table A1 reports the exact configuration used for every model architecture evaluated in Section 5.2 and every calibration method evaluated in Section 5.4. All models share the fixed seed (20260725), the 80/20 patient-level split, and the leakage-free preprocessing pipeline (Section 4.1); parameters not listed use the underlying library’s default value.

**Table A1:** Hyperparameter configuration by model. Feature-set ablation models (top) all use random_state=0; AutoGluon’s full configuration is detailed separately in Table A2.

Model	Configuration
Linear regression	scikit-learn LinearRegression, library defaults
Elastic Net	scikit-learn ElasticNet, library defaults (α=1.0, $\ell_{1}$ ratio =0.5)
Random Forest	scikit-learn RandomForestRegressor, n_estimators=300
MLP	scikit-learn MLPRegressor, hidden_layer_sizes=(64,32), max_iter=2000
Stacking ensemble	scikit-learn StackingRegressor; base estimators: linear regression, random forest (n_estimators=200), MLP (hidden_layer_sizes=(32,), max_iter=2000); final estimator: linear regression
XGBoost	XGBRegressor, n_estimators=300, tree_method=hist, GPU where available
CatBoost	CatBoostRegressor, native categorical handling (cat_features), GPU where available; missing genotype filled with the literal string "missing" rather than imputed
TabPFN	TabPFNRegressor (Prior-Labs TabPFN-3), in-context learning, no hyperparameter search by design
MAPIE (conformal)	CrossConformalRegressor (MAPIE ≥ 1.4 API), α=0.10, wrapped around the fitted stacking ensemble
NGBoost	NGBRegressor, predictive-distribution interval, 90% nominal target coverage
Deep ensemble	5 MLPs, identical architecture, 5 independent random seeds; 90%-nominal interval = mean ±1.645× inter-model standard deviation

## Appendix D. RAG Explanation Prompt Template

The prototype interface’s Phase 9 RAG explanation layer (Section 6.4) constructs its LLM prompt from a fixed template, populated per patient with that prediction’s own SHAP attributions, the retrieved guideline passages, and the ancestry calibration-coverage note when non-empty. The template is reproduced verbatim below (curly-brace placeholders are filled in at request time; they are not sent to the model as literal text).

You are explaining a warfarin dose prediction to a clinician.
You MUST base every factual claim ONLY on the SHAP feature
attributions and retrieved passages given below.
For every sentence that makes a factual claim, cite its source
in brackets, e.g., [SHAP: VKORC1] or [CPIC 2017 Warfarin
Guideline (rs9923231)].
Do NOT introduce any medical fact that is not present in the
inputs below, even if you believe it to be true.
If the ancestry coverage note below is non-empty, include it
verbatim as the final sentence.

Predicted Stable Weekly Dose: {weekly_dose} mg/week
  (Daily: {daily_dose} mg/day)
90% Conformal Prediction Interval:
  ({interval_low} - {interval_high}) mg/week

SHAP feature attributions (feature: contribution):
{shap_lines}

Retrieved Guideline Passages:
{passage_lines}

Ancestry coverage note (include verbatim as the final sentence
  if non-empty): {ancestry_note}

Explanation:

Two distinct controls are in place, one preventive and one detective, and together they cover both ways a claim can go wrong. The preventive control is the prompt itself, reproduced in full above: it instructs the model to base every claim only on the supplied SHAP attributions and passages and explicitly forbids introducing any fact not present in them, constraining both an unsupported claim and an inaccurate one before generation, though it cannot guarantee either is avoided. The detective control acts after generation, by regex-extracting every bracketed citation the model returns and checking it in two ways against what was actually supplied in this prompt (Section 6.4): first, that the citation’s source, a SHAP feature name or a retrieved passage, actually exists among those supplied for that query, which catches an *unsupported* claim, a citation pointing to a source never actually supplied; second, for a SHAP citation specifically, that the mg/week magnitude and direction stated in the same sentence match that feature’s real signed contribution within a fixed tolerance, which catches an *inaccurate* claim attached to a real citation, for example, a correct feature name with a fabricated direction or magnitude. A passage citation is not checked this second way, since a retrieved passage’s content is free text, not a single number to compare against. Either failure is flagged in the interface as a visible audit count, color-coded, rather than presented as equally grounded. When no generation backend is configured at all, a deterministic, template-based generator built from the same verified sources is used instead, so an explanation is always available and always checked rather than silently absent or unchecked.

## Appendix E. AutoGluon Configuration Details

Table A2 summarizes the configuration and resulting artifacts of the winning model on both cohorts.

**Table A2:** AutoGluon configuration and resulting artifacts, IWPC-6256 and IWPC-1780.

Parameter	Value
*Base configuration*	
Framework	AutoGluon Tabular
Preset	extreme_quality
Time limit (per model)	600 s
Portfolio size	≈22 base models
*Excluded model types*	
TABDPT, TABICL, TABM, MITRA (Section 4.3)	
*Final ensemble composition*	
IWPC-6256 winner	WeightedEnsemble_L2 (RealTabPFN-v2_c1, weight 1.0)
IWPC-1780 winner	WeightedEnsemble_L2 (RealTabPFN-v2_r13 + LightGBMPrep_r13 + RealTabPFN-v2_r106)
*Hugging Face artifacts (public)*	
https://huggingface.co/HassanB4/warfarisk-autogluon-6256 (HassanB4/warfarisk-autogluon-6256) (accessed on 8 September 2026)	
https://huggingface.co/HassanB4/warfarisk-autogluon-1780 (HassanB4/warfarisk-autogluon-1780) (accessed on 8 September 2026)	
Verified via	HfApi.repo_info(), 25 July 2026; model cards updated and repositories made public, 10 August 2026

## References

[1] International Warfarin Pharmacogenetics Consortium (2009). Estimation of the Warfarin Dose with Clinical and Pharmacogenetic Data. N. Engl. J. Med..

[2] Gage B.F., Eby C., Johnson J.A., Deych E., Rieder M.J., Ridker P.M., Milligan P.E., Grice G., Lenzini P., Rettie A.E. (2008). Use of Pharmacogenetic and Clinical Factors to Predict the Therapeutic Dose of Warfarin. Clin. Pharmacol. Ther..

[3] Wang M., Qian Y., Yang Y., Chen H., Rao W.F. (2024). Improved Stacking Ensemble Learning Based on Feature Selection to Accurately Predict Warfarin Dose. Front. Cardiovasc. Med..

[4] Fülöp P., Tóth Š., Porubán T., Fülöpová Z., Borovská A., Dvorožňáková M. (2025). Machine Learning for Warfarin Therapy: A Systematic Review. Pharmaceuticals.

[5] Johnson J.A., Caudle K.E., Gong L., Whirl-Carrillo M., Stein C.M., Scott S.A., Lee M.T.M., Gage B.F., Kimmel S.E., Perera M.A. (2017). Clinical Pharmacogenetics Implementation Consortium (CPIC) Guideline for Pharmacogenetics-Guided Warfarin Dosing: 2017 Update. Clin. Pharmacol. Ther..

[6] Cosgun E., Limdi N.A., Duarte C.W. (2011). High-Dimensional Pharmacogenetic Prediction of a Continuous Trait Using Machine Learning Techniques with Application to Warfarin Dose Prediction in African Americans. Bioinformatics.

[7] Perera M.A., Cavallari L.H., Limdi N.A., Gamazon E.R., Konkashbaev A., Daneshjou R., Pluzhnikov A., Crawford D.C., Wang J., Liu N. (2013). Genetic Variants Associated with Warfarin Dose in African-American Individuals: A Genome-Wide Association Study. Lancet.

[8] Drozda K., Wong S., Patel S.R., Bress A.P., Nutescu E.A., Kittles R.A., Cavallari L.H. (2015). Poor Warfarin Dose Prediction with Pharmacogenetic Algorithms That Exclude Genotypes Important for African Americans. Pharmacogenet. Genom..

[9] Gottlieb A., Daneshjou R., DeGorter M., Bourgeois S., Svensson P.J., Wadelius M., Deloukas P., Montgomery S.B., Altman R.B. (2017). Cohort-Specific Imputation of Gene Expression Improves Prediction of Warfarin Dose for African Americans. Genome Med..

[10] Zhang H., Alarcon C., Cavallari L.H., Nutescu E., Carvill G.L., Perera M.A., Hernandez W. (2023). Genomewide Association Study Identifies Copy Number Variants Associated with Warfarin Dose Response and Risk of Venous Thromboembolism in African Americans. Clin. Pharmacol. Ther..

[11] Lindley K.J., Limdi N.A., Cavallari L.H., Perera M.A., Lenzini P., Johnson J.A., Wu A.H., Ridker P.M., King C.R., Eby C.S. (2022). Warfarin Dosing in Patients with CYP2C9*5 Variant Alleles. Clin. Pharmacol. Ther..

[12] Zeng J., Shao J., Lin S., Zhang H., Su X., Lian X., Zhao Y., Ji X., Zheng Z. (2022). Optimizing the dynamic treatment regime of in-hospital warfarin anticoagulation in patients after surgical valve replacement using reinforcement learning. J. Am. Med. Inform. Assoc..

[13] Anzabi Zadeh S., Street W.N., Thomas B.W. (2023). Optimizing warfarin dosing using deep reinforcement learning. J. Biomed. Inform..

[14] Jahmunah V., Chen S., Oh S.L., Acharya U.R., Chowbay B. (2023). Automated Warfarin Dose Prediction for Asian, American, and Caucasian Populations Using a Deep Neural Network. Comput. Biol. Med..

[15] Dryden L., Song J., Valenzano T.J., Yang Z., Debnath M., Lin R., Topolovec-Vranic J., Mamdani M., Antoniou T. (2023). Evaluation of Machine Learning Approaches for Predicting Warfarin Discharge Dose in Cardiac Surgery Patients: Retrospective Algorithm Development and Validation Study. JMIR Cardio.

[16] Petch J., Nelson W., Wu M., Ghassemi M., Benz A., Fatemi M., Di S., Carnicelli A., Granger C., Giugliano R. (2024). Optimizing warfarin dosing for patients with atrial fibrillation using machine learning. Sci. Rep..

[17] Taquet V., Blot V., Morzadec T., Lacombe L., Brunel N. (2022). MAPIE: An Open-Source Library for Distribution-Free Uncertainty Quantification. arXiv.

[18] Lundberg S.M., Lee S.I. A Unified Approach to Interpreting Model Predictions. Proceedings of the Advances in Neural Information Processing Systems.

[19] Nori H., Jenkins S., Koch P., Caruana R. (2019). InterpretML: A Unified Framework for Machine Learning Interpretability. arXiv.

[20] Lou Y., Caruana R., Gehrke J. Intelligible Models for Classification and Regression. Proceedings of the 18th ACM SIGKDD International Conference on Knowledge Discovery and Data Mining.

[21] Obermeyer Z., Powers B., Vogeli C., Mullainathan S. (2019). Dissecting Racial Bias in an Algorithm Used to Manage the Health of Populations. Science.

[22] Rajkomar A., Hardt M., Howell M.D., Corrado G., Chin M.H. (2018). Ensuring Fairness in Machine Learning to Advance Health Equity. Ann. Intern. Med..

[23] McDermott M.B.A., Wang S., Marinsek N., Ranganath R., Foschini L., Ghassemi M. (2021). Reproducibility in Machine Learning for Health Research: Still a Ways to Go. Sci. Transl. Med..

[24] Collins G.S., Moons K.G.M., Dhiman P., Riley R.D., Beam A.L., Van Calster B., Ghassemi M., Liu X., Reitsma J.B., van Smeden M. (2024). TRIPOD+AI Statement: Updated Guidance for Reporting Clinical Prediction Models That Use Regression or Machine Learning Methods. BMJ.

[25] Moons K.G.M., Damen J.A.A., Kaul T., Hooft L., Andaur Navarro C., Dhiman P., Beam A.L., Van Calster B., Celi L.A., Denaxas S. (2025). PROBAST+AI: An Updated Quality, Risk of Bias, and Applicability Assessment Tool for Prediction Models Using Regression or Artificial Intelligence Methods. BMJ.

[26] Fung E., Patsopoulos N.A., Belknap S.M., O’Rourke D.J., Robb J.F., Anderson J.L., Shworak N.W., Moore J.H. (2012). Effect of genetic variants, especially *CYP2C9* and *VKORC1*, on the pharmacology of warfarin. Semin. Thromb. Hemost..

[27] Johnson A.E.W., Pollard T.J., Shen L., Lehman L.W.H., Feng M., Ghassemi M., Moody B., Szolovits P., Celi L.A., Mark R.G. (2016). MIMIC-III, a Freely Accessible Critical Care Database. Sci. Data.

[28] Johnson A.E.W., Bulgarelli L., Shen L., Gayles A., Shammout A., Horng S., Pollard T.J., Hao S., Moody B., Gow B. (2023). MIMIC-IV, a Freely Accessible Electronic Health Record Dataset. Sci. Data.

[29] Pollard T.J., Johnson A.E.W., Raffa J.D., Celi L.A., Mark R.G., Badawi O. (2018). The eICU Collaborative Research Database, a Freely Available Multi-Center Database for Critical Care Research. Sci. Data.

[30] Wani A.A., Abeer F. (2025). Application of machine learning techniques for warfarin dosage prediction: A case study on the MIMIC-III dataset. PeerJ Comput. Sci..

[31] Truda G., Marais P. (2021). Evaluating Warfarin Dosing Models on Multiple Datasets with a Novel Software Framework and Evolutionary Optimisation. J. Biomed. Inform..

[32] Prokhorenkova L., Gusev G., Vorobev A., Dorogush A.V., Gulin A. CatBoost: Unbiased Boosting with Categorical Features. Proceedings of the Advances in Neural Information Processing Systems.

[33] Hollmann N., Müller S., Purucker L., Krishnakumar A., Körfer M., Hoo S.B., Schirrmeister R.T., Hutter F. (2025). Accurate Predictions on Small Data with a Tabular Foundation Model. Nature.

[34] Erickson N., Mueller J., Shirkov A., Zhang H., Larroy P., Li M., Smola A. (2020). AutoGluon-Tabular: Robust and Accurate AutoML for Structured Data. arXiv.

[35] Duan T., Avati A., Ding D.Y., Thai K.K., Basu S., Ng A.Y., Schuler A. NGBoost: Natural Gradient Boosting for Probabilistic Prediction. Proceedings of the 37th International Conference on Machine Learning.

[36] Ma W., Li H., Dong L., Zhou Q., Fu B., Hou J.L., Wang J., Qin W., Chen J. (2021). Warfarin maintenance dose prediction for Chinese after heart valve replacement by a feedforward neural network with equal stratified sampling. Sci. Rep..

[37] Asiimwe I.G., Blockman M., Cohen K., Cupido C., Hutchinson C., Jacobson B., Lamorde M., Morgan J., Mouton J.P., Nakagaayi D. (2022). Stable warfarin dose prediction in sub-Saharan African patients: A machine-learning approach and external validation of a clinical dose-initiation algorithm. CPT Pharmacomet. Syst. Pharmacol..

[38] Steiner H.E., Giles J.B., Patterson H.K., Feng J., El Rouby N., Claudio K., Marcatto L.R., Tavares L.C., Galvez J.M., Calderon-Ospina C.A. (2021). Machine Learning for Prediction of Stable Warfarin Dose in US Latinos and Latin Americans. Front. Pharmacol..

[39] Kimmel S.E., French B., Kasner S.E., Johnson J.A., Anderson J.L., Gage B.F., Rosenberg Y.D., Eby C.S., Madigan R.A., McBane R.B. (2013). A Pharmacogenetic versus a Clinical Algorithm for Warfarin Dosing. N. Engl. J. Med..

[40] Pirmohamed M., Burnside G., Eriksson N., Jorgensen A.L., Toh C.H., Nicholson T., Kesteven P., Christersson C., Wahlström B., Stafberg C. (2013). A Randomized Trial of Genotype-Guided Dosing of Warfarin. N. Engl. J. Med..

[41] Gage B.F., Bass A.R., Lin H., Woller S.C., Stevens S.M., Al-Hammadi N., Li J., Rodriguez T., Miller J.P., McMillin G.A. (2017). Effect of Genotype-Guided Warfarin Dosing on Clinical Events and Anticoagulation Control Among Patients Undergoing Hip or Knee Arthroplasty: The GIFT Randomized Clinical Trial. JAMA.

[42] Whirl-Carrillo M., Huddart R., Gong L., Sangkuhl K., Thorn C.F., Whaley R., Klein T.E. (2021). An Evidence-Based Framework for Evaluating Pharmacogenomics Knowledge for Personalized Medicine. Clin. Pharmacol. Ther..

[43] Sellergren A., Kazemzadeh S., Jaroensri T., Kiraly A., Traverse M., Kohlberger T., Xu S., Jamil F., Hughes C., Lau C. (2025). MedGemma Technical Report. arXiv.

[44] Borishetty D., Banda P., Andhi N., Desai A., Uppili B.R., Samudrala N.M., Palanki S.S., Bobbili D.R., Iyer G.R. (2025). PGxRAG: A Retrieval Augmented Generation Supported Pharmacogenomics Assistant. medRxiv.

[45] El Rouby N., Shahin M.H., Bader L., Khalifa S.I., Elewa H. (2022). Genomewide association analysis of warfarin dose requirements in Middle Eastern and North African populations. Clin. Transl. Sci..

[46] Alqurain A., Alshridha T.I., Al-Otaibi S., Al-Otaibi D.F., Douba F.M., Aldhobaib A.I., Alammar N., Khubrani M.M., Althobaiti W., Alwashmi R.M. (2026). Clinical Pharmacogenomic Variants Among the Saudi Population and Their Impact on Drug Response: A Review of Saudi-Based Evidence. Cureus.

[47] Jokhab S., AlRasheed M.M., Bakheet D., AlMomen A., AlAboud N., Kamali F. (2025). The impact of CYP2C9, VKORC1, and CYP4F2 polymorphisms on warfarin dose requirement in Saudi patients. Front. Pharmacol..

[48] Kuang Y., Liu Y., Pei Q., Ning X., Zou Y., Liu L., Song L., Guo C., Sun Y., Deng K. (2022). Long Short-Term Memory Network for Development and Simulation of Warfarin Dosing Model Based on Time Series Anticoagulant Data. Front. Cardiovasc. Med..

